# The Influence of Photoperiod on the Action of Exogenous Leptin on Gene Expression of Proinflammatory Cytokines and Their Receptors in the Thoracic Perivascular Adipose Tissue (PVAT) in Ewes

**DOI:** 10.1155/2019/7129476

**Published:** 2019-11-12

**Authors:** Agata Krawczyńska, Andrzej P. Herman, Hanna Antushevich, Joanna Bochenek, Karolina Wojtulewicz, Dorota A. Zięba

**Affiliations:** ^1^The Kielanowski Institute of Animal Physiology and Nutrition, Polish Academy of Sciences, Instytucka 3, 03-105 Jabłonna, Poland; ^2^Department of Animal Biotechnology, Laboratory of Biotechnology and Genomics, Agricultural University of Krakow, 30-248 Krakow, Poland

## Abstract

Leptin resistance is either a condition induced by human obesity or a natural phenomenon associated with seasonality in ruminants. In the cardiovascular system, the leptin resistance state presence is a complex issue. Moreover, the perivascular adipose tissue (PVAT) appears to be crucial as a source of proinflammatory cytokines and as a site of interaction for leptin contributing to endothelium dysfunction and atherosclerosis progression. So the aim of this study was to examine the influence of the photoperiod on the action of exogenous leptin on gene expression of selected proinflammatory cytokines and their receptors in thoracic PVAT of ewe with or without prior lipopolysaccharide (LPS) stimulation. The experiment was conducted on 48 adult, female ewes divided into 4 group (*n* = 6 in each): control, with LPS intravenous (iv.) injection (400 ng/kg of BW), with leptin iv. injection (20 *μ*g/kg BW), and with LPS and 30-minute-later leptin injection, during short-day (SD) and long-day (LD) seasons. Three hours after LPS/control treatment, animals were euthanized to collect the PVAT adherent to the aorta wall. The leptin injection enhanced *IL1B* gene expression only in the LD season; however, in both seasons leptin injection intensified LPS-induced increase in *IL1B* gene expression. *IL1R2* gene expression was increased by leptin injection only in the SD season. Neither *IL6* nor its receptor and signal transducer gene expressions were influenced by leptin administration. Leptin injection increased *TNFA* gene expression regardless of photoperiodic conditions. Only in the SD season did leptin treatment increase the gene expression of both TNF*α* receptors. To conclude, leptin may modulate the inflammatory reaction progress in PVAT. In ewe, the sensitivity of PVAT on leptin action is dependent upon the photoperiodic condition with stronger effects stated in the SD season.

## 1. Introduction

Perivascular adipose tissue (PVAT), the adipose tissue surrounding vessels, was primarily believed to play a structural role in protecting vessels during contraction. Now it is well documented that PVAT is also a crucial regulator of vascular function [1]. Being located in close contact with fibroblasts, vascular smooth muscle cells, or endothelial cells, PVAT can act in the endocrine or the paracrine way secreting adipokines (e.g., leptin, adiponectin, and resistin), cytokines, chemokines, etc. [2]. It is believed that anatomically, PVAT varies greatly depending on the location. The thoracic aorta is surrounded mostly by brown PVAT (thoracic PVAT); further on, the beige PVAT is present around the abdominal aorta (abdominal PVAT); and finally, the white PVAT surrounds the small arteries (mesenteric PVAT) [1, 3]. The high thoracic PVAT content was found to be significantly associated with a higher prevalence of cardiovascular disease (CVD) [4]. In the pathological states, such as obesity or diabetes, PVAT can also be a significant source of an excessive amount of proinflammatory cytokines contributing to endothelium dysfunction and atherosclerosis progression [5]. Regardless the mediators of inflammation, such as interleukin (IL)-1*β*, IL6, or tumour necrosis factor (TNF)*α*, PVAT can also secrete a number of adipokines possessing similar proinflammatory properties such as leptin. Basically, leptin is supplied to the vessel walls from the lumen, but in pathological states, the leptin synthesis and secretion from PVAT may also be intensified. Leptin secreted by PVAT acts directly on the vessel as there is no anatomical barrier between the adventitia and adipocytes; that fact enables leptin to reach the smooth muscle cells more quickly [6, 7]. The action of leptin in vessels has a dual nature. As was demonstrated by Sikka et al. [8], leptin is essential to maintain the proper functioning of the blood vessels, regardless of body weight. Besides stimulating nitrite oxide (NO) synthesis, leptin acts as a vasodilator stimulating the endothelium-derived hyperpolarizing factor (EDHF) and production of hydrogen peroxide (H_2_O_2_) by endothelial cells [9]. Leptin receptors were also identified in both vascular smooth muscle cells [10] and endothelial cells [11]. There is no evidence for their presence in the adventitia, the least explored layer of the blood vessels; however, as was shown in our previous immunohistochemical analysis, the leptin protein is also present in this aortic layer, which indirectly may indicate its activity in this part of the vessel wall [12, 13]. On the other hand, leptin also appears to play an important role in the promotion of atherosclerotic lesions, because, as was stated by Schroeter et al. [14], leptin receptors are present in the atherosclerotic plaque. Moreover, ob/ob mice (with leptin gene knockout) are resistant to atherosclerosis [15]. It is believed that in the pathological conditions (e.g., obesity), leptin accelerates the development of atherosclerosis by several mechanisms: increasing synthesis of proinflammatory cytokines in macrophages and monocytes (IL6, IL12, IL18, and TNF*α*), enhancing expression of endothelin 1, intensifying oxidative stress in endothelial cells, promoting migration and proliferation of the vascular smooth muscle cell, and stimulating platelet aggregation [16]. Also, NO-dependent vasorelaxing actions of leptin are impaired as a result of selective vascular leptin resistance. Leptin resistance is a state when the cells/organs are insensitive to elevated levels of leptin. The main effect is observed in the brain where leptin actions are strictly connected with energy metabolism and food intake. In vessels, the leptin resistance means inability to obtain the NO-mimetic vasorelaxing effect of leptin in contrast to its vasoconstricting effects. As it was observed by Bełtowski et al. [17, 18], acute administration of leptin in lean rats elevated the level of NO metabolites and cyclic guanosine monophosphate (cGMP) in plasma and the aortic wall, but the acute effects of leptin were impaired in animals with hyperleptinemia caused by both high-caloric “cafeteria diet” or chronic leptin injection. Therefore, the existence of vessel leptin resistance in the cardiovascular system in pathological conditions is confirmed.

The obesity-induced leptin resistance has been the interest of scientists for several years. It encompasses a complex pathophysiological phenomenon with a number of potential research lines in terms of its mechanisms or diagnostics. Connected with the presence of hormonal imbalance, reproductive disturbance, insulin resistance, diabetes, or hypertension, leptin resistance is a rather negative state in humans [19]. However, in ewe, the phenomenon of natural leptin resistance is observed in the long-day season (spring/summer), which is connected with seasonal adaptation to changes in energy supply and demand. Szczesna and Zieba [20] concentrated on the central leptin resistance, which can explain the increased food intake with simultaneous high blood leptin level in the long-day season. The existence of leptin resistance or the changes in leptin sensitivity in the peripheral tissue of ewe in different photoperiodic conditions have not been examined yet; also, the possible importance of the presence of such a state, for example, in ewes' vessels, was not discussed before.

The aim of this study was to examine the influence of photoperiodic conditions (long-day (LD) and short-day (SD) seasons) on the action of exogenous leptin on gene expression of proinflammatory interleukins and their receptors in PVAT of ewes with or without prior acute inflammation induction.

## 2. Materials and Methods

### 2.1. Animals and Experimental Design

The procedures connected with animal welfare and caring were approved by the 3^rd^ Local Ethical Commission of Warsaw University of Life Sciences-SGGW (Warsaw, Poland, authorisation number 56/2013). The ewes were in good condition and were kept under constant veterinary care.

The experiment was conducted on 48 adult, about 2-year-old, female blackface ewes during two different photoperiods: in December (SD season; day : night 8 : 16) and in June (LD season; day : night 16 : 8). The animals were maintained indoors under natural lighting conditions (latitude 52°N, 21°E) in individual pens. The stress of social isolation was limited by visual contact with other members of the flock. The animals were acclimated to the experimental conditions for one month. The animals were fed a consistent diet of commercial concentrates with hay and water available *ad libitum* according to the recommendations of the National Research Institute of Animal Production (Krakow, Poland) [21].

In the SD season, the stage of the oestrous cycle of ewes was synchronized by the Chronogest® CR (Merck Animal Health, Boxmeer, The Netherlands) method using an intravaginal sponge impregnated with 20 mg of a synthetic progesterone-like hormone. All ewes had Chronogest® CR sponges placed for 14 days. After sponge removal, the ewes received an intramuscular injection of 500 iu of pregnant mare's serum gonadotropin (PMSG) (Merck Animal Health, Boxmeer, The Netherlands). The experimental procedure began 24 h following PMSG injection, so the ewes were in the follicular phase of the oestrous cycle. During the LD season, the animal synchronization was not required as animals were in seasonal anoestrous.

In both experiments, the animals were randomly divided into 4 groups, *n* = 6 in each: control (C), with LPS injection to induce immune stress (LPS), with leptin injection (LEP), and with LPS and leptin injection (LPS+LEP). The LPS from *Escherichia coli* 055:B5 (Sigma-Aldrich, St. Louis, MO, USA) was dissolved in saline and injected into the jugular vein at a dose of 400 ng/kg of body mass [22]. The recombinant sheep leptin (Protein Laboratories Rehovot (PLR) Ltd., Rehovot, Israel), also dissolved in saline, was injected 30 min after LPS treatment at a dose of 20 *μ*g/kg of body mass (based on the dose used for growing beef heifers according to Maciel et al. [23]). The used dose of leptin caused a significant increase in the leptin blood level up to 22 ng/mL 30 min after injection in the LEP group in the LD season (the basal leptin level was 1.19 ng/mL in this group and 0.44 ng/mL in the LEP group in the SD season), which at the end of experiment decreased to 10.54 ng/mL (the data presenting leptin blood profile are in press). The control animals received an equivalent volume of saline (0.9% *w*/*v* NaCl; Baxter, Deerfield, IL, USA) at the moment of LPS and leptin injection. The experiment scheme is presented in Table 1.

Three hours after LPS/saline treatment (which was 2.5 h after leptin/saline injection), the animals were euthanized and samples of PVAT adherent to the thoracic aorta wall were collected. All tissues were washed in saline, frozen in liquid nitrogen, and stored at −80°C.

### 2.2. Relative mRNA Expression

Total RNA from the PVAT was isolated using a RIBOZOL (VWR Chemicals, Solon, OH, USA) according to the manufacturer's instruction. The quantity and quality of total RNA were quantified spectrophotometrically at 260 and 280 nm with the use of a NanoDrop 1000 instrument (Thermo Fisher Scientific Inc., Waltham, MA, USA). The RNA integrity was checked by 1% agarose gel electrophoresis. The cDNA synthesis was performed using a Maxima™ First Strand cDNA Synthesis Kit for RT-qPCR (Thermo Fisher Scientific Inc., Waltham, MA, USA) according to the manufacturer's instruction. 1200 ng of total RNA was used as starting material for reversed transcription in reaction volume of 20 mL.

Real-time PCR assay was carried out with the use of a 5x HOT FIREPol EvaGreen qPCR Mix Plus (no ROX) (Solis BioDyne, Tartu, Estonia) and HPLC-grade oligonucleotide primers purchased from GenoMed (Warsaw, Poland). Specific primers for determining the expression of examined and reference genes are presented in Table 2. Each PCR reaction contained 3 *μ*L qPCR mix, 10 *μ*L RNase-free water, 0.225 *μ*L of each primer (working concentration 0.5 mM), and 1.5 *μ*L cDNA template (previously 3x diluted). The reactions were run on the Rotor-Gene Q thermocycler (Qiagen, Dusseldorf, Germany) using the following protocol: 95°C for 15 min and 35 cycles of 94°C for 5 s for denaturation, 59°C for 20 s for annealing, and 72°C for 5 s for extension. After the cycles, a final melting curve analysis with continuous fluorescence measurements was performed to confirm the specificity of the amplification.

The relative gene expression was calculated using the comparative quantification option of the Rotor-Gene Q Series Software 2.0.3 (Qiagen, Dusseldorf, Germany). To compensate variation in cDNA concentrations and PCR efficiency between samples, an endogenous control gene was amplified in each sample and used for normalization. Initially, three reference genes (*HDAC1*, *ACTB*, and *GAPDH*) were tested; however, after analysis with the use of NormFinder software [28], the *HDAC1* gene was stated as the endogenous control with the best expression stability in the experimental design. The results are presented in arbitrary units, as the ratio of the target gene expression to the expression of the reference gene in the control group was calculated as 1.

### 2.3. Statistical Analysis

Statistical analysis was performed using STATISTICA v. 13.1 (Dell Inc., Round Rock, TX, USA). Results of two-way (LPS and leptin injection) analysis of variance (ANOVA) followed by *post hoc* Tukey's test were considered statistically significant at *P* ≤ 0.05. The results for each season were analysed separately. The ANOVA analysis was performed after its two assumptions, normality (Shapiro-Wilk's test) and homogeneity of variances (Levene's test), were checked. The *post hoc* test was performed only if one of the main factors exerted a significant effect according to the ANOVA test. All data are presented as means ± standard deviation (SD).

## 3. Results

### 3.1. Leptin Receptor (Figure 1)

In the SD season, Tukey's *post hoc* test showed that in comparison to the control group, the *LEPR* gene expression was increased after leptin injection (Tukey's test, C vs. LEP, *P* ≤ 0.001). Moreover, the higher *LEPR* mRNA levels were observed in the LEP and LPS+LEP groups in comparison to the LPS group (Tukey's test, LPS vs. LEP and LPS+LEP, *P* ≤ 0.0001 and *P* ≤ 0.001, respectively). In the LD season, both the LPS and leptin injections decreased *LEPR* gene expression (ANOVA, *P* ≤ 0.0001 for LPS effect and *P* ≤ 0.05 for leptin effect). The lowest *LEPR* mRNA level was stated for the LPS group, while the LEP and LPS+LEP groups did not differ from each other but were still lower than the control one.

### 3.2. Interleukin-1*β* and Its Receptors (Figure 2)

The obtained results showed that regardless the season, the LPS injection increased the gene expression of *IL1B* and both its receptors (*IL1R1* and *IL1R2*) (ANOVA, *P* ≤ 0.0001 for all three genes in both seasons), but in the SD season, the influence of endotoxin on the *IL1R2* gene expression was more pronounced (8.87-fold change vs. 5.31-fold change in SD and LD season, respectively).

The single leptin injection enhanced the *IL1B* gene expression only in the LD season (Tukey's test, C vs. LEP, *P* ≤ 0.02); however, in both seasons, leptin injection intensified the LPS-induced increase in *IL1B* gene expression (Tukey's test, LPS vs. LPS+LEP, *P* ≤ 0.001 and *P* ≤ 0.002 for the SD and LD seasons, respectively). Leptin injection did not influence *IL1R1* mRNA levels. The *IL1R2* gene expression was increased by leptin injection only in the SD season (Tukey's test, C vs. LEP, *P* ≤ 0.0001), whereas no additive effect of LPS and leptin was observed on this gene expression.

### 3.3. Interleukin-6, Its Receptor, and Signal Transducer (Figure 3)

There was observed an effect of endotoxin injection on *IL6*, its receptor (*IL6R*), and its signal transducer (*IL6ST*) gene expressions in both seasons regardless leptin injection (ANOVA, *P* ≤ 0.0001 for all three genes in both seasons). The *IL6* and *IL6ST* gene expressions were enhanced, and *IL6R* was decreased after LPS injection. In the SD season, the influence of endotoxin on *IL6* was more pronounced (293-fold increase) than that on the LD one (115-fold increase).

Neither *IL6* nor its receptor and signal transducer gene expressions were influenced by leptin regardless of the photoperiodic conditions.

### 3.4. TNF*α* and Its Receptors (Figure 4)

*TNFA* gene expression was not influenced by singular LPS injection regardless the photoperiodic season. On the other hand, both *TNFR1* and *TNFR2* gene expressions were increased by LPS injection regardless the season (ANOVA, *P* ≤ 0.0001 for both genes in the two seasons).

Leptin injection increased *TNFA* gene expression regardless the photoperiodic conditions (ANOVA, *P* ≤ 0.0001 for both seasons); however, in the SD season, this effect was more pronounced (3.31-fold vs. 1.78-fold change in the SD and LD seasons, respectively). Only in the SD season did leptin injection increase the gene expression of both TNF*α* receptors (Tukey's test, C vs. LEP, *P* ≤ 0.002 and *P* ≤ 0.04 for *TNFR1* and *TNFR2*, respectively) and intensified the LPS-induced *TNFR2* gene expression (Tukey's test, LPS vs. LPS+LEP, *P* ≤ 0.0004).

## 4. Discussion

The fact that leptin is synthesized and secreted by adipose tissue is generally known; however, theories on leptin action on this tissue are inconsistent [29–31]. Basing on microarray profiling of human white adipose tissue (WAT), Taleb et al. [32] concluded that leptin can act on this tissue, especially on the expression of genes related to inflammation and immunity, but WAT structure and functions are very different from PVAT, especially the thoracic one. There is no literature data on whether intravenous leptin administration affects PVAT activity either in healthy organisms or in animals with induced systemic acute inflammation. Moreover, in the present study, the “long-day sheep,” a large animal model used as a model of the hyperleptinemic state, was used [33]. Moreover, Zieba et al. [34] even made a further suggestion proposing the “long-day ewe” as a model for obesity research because obese people as “long-day ewes” are characterized by enhanced food intake and reduced energy expenditure accompanied by a high leptin level. It is worth mentioning that sheep is also considered to be an accepted animal model in immunological studies because the sheep shows similar sensitivity to endotoxins to primates in contrast to rodents [35, 36]. Moreover, the fact that sheep, in contrast to the mouse and rat, is a diurnal animal also influences the immune response because immune system activity exhibits important oscillation over the course of a day [37]. The limited usefulness of small rodents as an animal model in experiments in the field of immunology was noticed by the USA Food and Drug Administration, which came to the conclusion that all new drugs developed to be used to treat the symptoms of systemic inflammation, before the start of clinical trials, must be tested on at least one recognized nonrodent animal model [35]. Considering the abovementioned information, we assumed that sheep would be an interesting model in the studies connecting leptin, inflammation, and photoperiod to show whether leptin can modulate the course of the inflammatory reaction in PVAT.

The seasonal changes in the *LEPR* gene expression in PVAT after LPS and leptin administration were observed. In SD season, the stimulating effect of leptin on its own receptor was stated regardless the immune status of animals. In contrast, in the LD season, both examined factors (LPS and leptin) decreased the *LEPR* expression. As the gene expression of the long form of leptin receptor (OB-Rb) was very low in the collected PVAT, the expression of the leptin receptor basing on the fragment of sequence encoding all kinds of leptin receptors (exons 6 and 7, whereas exon 20 is specific for each form, long and short one) was examined. As mentioned previously the “long-day ewe” is characterized by hyperleptinemia with impaired leptin action; such a state is mostly characteristic for obese people with impaired energy metabolism and food intake. But leptin resistance was also confirmed in the vessel where it results in an inability to obtain the NO-mimetic vasorelaxing effect of leptin in contrast to its vasoconstricting effects [17, 18]. The several mechanisms lying behind leptin vessel resistance have been proposed; however, many of them are based on the observation made according to neuronal actions of leptin, so there is a need for their verification in the arteries [38]. The downregulation of the leptin receptor, as a kind of leptin self-regulation, was observed in the hypothalamus of rats with diet-induced obesity [39]. Also, Bohlen et al. [40] stated that gene expression of OB-Rb decreased upon prolonged exposure to leptin in human aortic smooth muscle cells. On the other hand, in hyperleptinemic spontaneously hypertensive rats, the expression of leptin receptors is enhanced [41]. In the present study, the receptor expression after leptin injection is decreased in the LD season which can also be proposed as the mechanism of self-regulation in the conditions of hyperleptinemia. However, further investigations are needed to evaluate whether this is a sufficient mechanism to induce leptin resistance in PVAT. The molecular mechanism connected with a negative feedback mechanism of intracellular suppressor of cytokine signalling 3 (SOCS-3) on leptin Janus kinases (JAK)/signal transducer and activator of the transcription 3 (STAT3) pathway should be the next step, especially when Szczesna et al. [42] demonstrated that the photoperiod may influence leptin effects on the SOCS-3 expression in the sheep pituitary.

The next point in the presented study was to examine whether leptin influences proinflammatory cytokine gene expression in physiological conditions and if it modulates the progress of acute inflammation reaction. The impact of the photoperiod was also considered in this matter. Basing on the obtained results, it can be stated that intravenous injection of leptin increased the TNF*α* gene expression regardless the examined season and the presence of acute inflammation; however, only in the SD photoperiod did leptin administration also stimulate TNF*α* receptor gene expression. This may indicate that during the LD photoperiod, the leptin action is partially inhibited. This may also suggest increased sensitivity of PVAT to TNF*α* action during the SD season. The gene expression increased by exogenous leptin TNF*α* and its receptor suggests that leptin can influence the autocrine activity of TNF*α* on PVAT; however, TNF*α* may also migrate to the aorta wall because of direct contact with PVAT and adventitia and so exert effect over there. Generally, in the vessels, TNF*α* reduces NO bioavailability, induces oxidative stress and reactive oxygen substrates (ROS) formation, or increases proinflammatory cytokine synthesis, playing a significant role in vascular function impairment [43]. On the other hand, in adipose tissue, TNF*α* participates in the inhibition of carbohydrate metabolism (can induce a state of insulin resistance in adipocytes), lipogenesis, adipogenesis, and thermogenesis and stimulation of lipolysis [44]. It also influences endocrine functions of adipose tissue suppressing the production of adiponectin [45] or promoting leptin release from adipocytes [46, 47]. The mechanism of leptin influence on TNF*α* expression has already been examined but never in PVAT. Lee et al. [48] stated that leptin increases TNF*α* in Raw 264.7 cells. The authors also suggested the possible pathway: phospholipase C (PLC*γ*)/Src/phospholipase D1 (PLD1)/phosphatidic acid (PA)/p70S6K/c-jun N-terminal protein kinase (JNK). They stated that leptin enhanced the activity of PLD1 through activation of PLC*γ* and Src, while PLD1 siRNA decreased the leptin-induced expression and production of TNF*α*. Leptin-induced PLD activation was also inhibited by a PLC*γ* inhibitor (POA) and Src kinase inhibitor (PP2) indicating PLC*γ* and Src kinase are upstream activators of PLD1. Earlier Shen et al. [49] showed that leptin can enhance TNF*α via* JNK and p38 MAPK pathways in LPS-stimulated Kupffer cells. In contrast, in the present study, leptin administration led to an increase in *TNFA* gene expression in PVAT regardless the prior LPS-stimulation and examined photoperiod. However, we did not observe a stimulatory effect of LPS on *TNFA* gene expression, although the same dose of LPS used in ewe increased *TNFA* in the hypothalamus [25]. Only in the SD season, the *TNFA* gene expression was the highest in the group treated with both LPS and leptin. It must be stressed that also only in this season did leptin injection increase the TNF*α* receptor gene expression in PVAT, which may show that especially in this season, the leptin-increased *TNFA* gene expression is associated with increased TNF*α* autocrine activity on this tissue. TNF*α* mediates its biological effects on adipose tissue *via* two distinct cell surface receptors: TNFR1 and TNFR2. The circulating levels of both these receptors are increased in both obesity and nonobesity adults with proatherogenic lipid profiles [50, 51]; however, it is TNFR1 that is said to be dominant. For example, the lack of TNFR1, but not the lack of TNFR2, significantly improves insulin sensitivity in ob/ob mice and cultured adipocytes [52, 53]. In the present study, the effect of season was stated for both *TNFR1* and *TNFR2*, which may suggest that both these receptors play a crucial role in mediating TNF*α* effects in a season-dependent manner. Moreover, the expression of *TNFR2* was synergistically increased by LPS and leptin, whereas in the case of *TNFR1*, no such effect was stated. The lack of leptin effect on TNF*α* receptor gene expression in the LD photoperiod with a simultaneous effect on *TNFA* gene expression may be considered in two ways. Firstly, it may be suggested that although *TNFA* content increases after leptin injection, TNF*α* action is not so pronounced that its receptor gene expression is not stimulated. It must be pointed out here that, as TNF*α* actions on adipose tissue are rather negative, such a mechanism might be regarded as a positive one. However, on the other hand, it cannot be excluded that during LD season, the basal protein expression of TNF*α* receptors is so high that further gene expression increase is not necessary. Further research, especially at the protein level, is required to clarify the existing mechanism.

In addition to the effect of leptin on TNF*α* activity in PVAT, injection of leptin also affected other studied proinflammatory cytokines. It was found that leptin administration potentates the stimulatory effect of LPS on *IL1B* gene expression in PVAT, but the individual effect of leptin on *IL1B* gene expression was stated only in the LD season. IL1B is one of the most potent proinflammatory cytokines promoting vascular inflammation. IL1B acts as not only a local vascular but also a systemic contributor in atherosclerosis progression [54]. Acting mainly by nuclear factor kappa B (NF-*κ*B) signalling, JNK, and p38 mitogen-activated protein kinase pathways [55], IL1B promotes the expression of other cytokines (e.g., IL6), adhesion molecules, and the migration and mitogenesis of the vascular smooth muscle [56]. Considering such negative effects of IL1B, the inhibition of its activity has become a potential therapeutic target in the prevention and treatment of atherosclerosis. The Canakinumab Anti-Inflammatory Thrombosis Outcome Study (CANTOS) was one of the clinical studies that concentrated on blocking the IL1B proinflammatory activity for atherosclerosis therapy [54]. In CANTOS, a human monoclonal antibody, canakinumab, was examined. In contrast to the IL1 receptor antagonist (IL1ra, anakinra), canakinumab acting selectively on IL1B but not on IL1A did not affect the host defences and susceptibility to the infection to such a large extent [54]. Although there are no works presenting the leptin effect on cytokine (and so IL1B) synthesis in sheep tissue, there are several works conducted on rats, however not in the context of PVAT. Luheshi et al. [57] stated that leptin increased Il1B in the hypothalamus of rats; furthermore, leptin action on appetite and body temperature was abolished by the IL1ra or in mice lacking the IL1 receptor. Next, Sachot et al. [58] showed that leptin is a circulating mediator of LPS-induced anorexia and fever probably through a hypothalamic IL1B-dependent mechanism (but not the IL6-dependent one) as fever and anorexia were attenuated in the presence of leptin antiserum. Moreover, Hosoi et al. [59] confirmed their thesis that leptin regulates IL1B expression in the brain *via* the STAT3-independent mechanisms conducting research on db/db rodents, which do not possess the active long form of the leptin receptor. As was mentioned above, IL1B effects are strictly connected with its receptor type I and II presence (IL1R1 and IL1R2, respectively). However, in the present study, we did not state the effect of leptin on *IL1R1* expression; the expression of *IL1R2* was increased by leptin only in the SD season. It should be stressed that although IL1B can be bound by two receptors, only IL1R1 is able to transduce the signal to the inside of the cell. IL1R2 acts as a decoy receptor and reduces the amount of substrate for the appropriate receptor - so its function can be treated as anti-inflammatory [60]. Considering the increased *IL1B* expression in LD and increased *IL1R2* expression in the SD season, it can be suggested that the leptin-induced IL1B activity may be more potent in the LD season, which is in contrast to the results stated for TNF*α* in the present study. However, regardless the season, leptin can enhance the stimulatory effect of LPS on *IL1B*, which stresses the proinflammatory activity of leptin in the PVAT of ewes with induced acute inflammation.

Adipose tissue is said to be one of the major sources of IL6 in the organism, producing 10–35% of circulating IL6 plasma levels in humans [61]. IL6 exerts pleiotropic actions in the organism; among them is the induction of symptoms that accompany infection, such as increased temperature. IL6 is even proposed as a new biomarker for the diagnosis of sepsis, which might be helpful to provide adequate and timely management of critically ill patients and thus reduce the morbidity and mortality associated with this condition [62]. Also in the cardiovascular system disease, IL6 is an upstream inflammatory cytokine that plays a central role in propagating the downstream inflammatory response responsible for atherosclerosis [63]. The usage of the IL6 inhibitor, tocilizumab, improved endothelial function and decreased aortic stiffness [64]. In the laboratory conditions, induction of inflammation with the use of LPS also significantly increases the IL6 plasma level as well as this interleukin gene expression in tissues [25]. In the present study, the increase in *IL6* gene expression after LPS injection was 293-fold whereas for *IL1B* only 3-fold. The seasonal differences in LPS effects on *IL6* gene expression in sheep PVAT are also interesting. In the SD season, the influence of endotoxin on *IL6* was more pronounced (293-fold increase) than that in the LD one (115-fold increase), which shows that response to LPS injection is season-dependent with higher sensitivity in the SD season. The explanation to such a condition could be connected with seasonal leptin resistance (lower sensitivity in the LD season); however, in the present study, no effect of leptin in both physiological and acute inflammation conditions was stated on IL6 and its receptor. It is rather surprising, especially when IL6 is said to be one of the most important proinflammatory cytokines secreted by adipose tissue and it is known (basing on studies conducted on rats) that sickness behaviour after LPS injection is mediated by both leptin and IL6 [65]. In contrast, Taleb et al. [32] stated that a single supraphysiological dose of polyethylene glycol-leptin injected to healthy nonobese men can decrease *IL6* gene expression in WAT 72 h after treatment. The anti-inflammatory effect of leptin, but in plasma, was stated by Xiao et al. [66] who observed that leptin infusion before endotoxin treatment attenuated IL6 and cortisol responses to the endotoxin in ovariectomized rhesus monkeys. They concluded that leptin can be both released in response to inflammation and act to attenuate the response to proinflammatory cytokines. Such conclusions are in contrast to the results obtained in this study, which could be due to differences in animal model (ovariectomized animals vs. females with active ovaries but synchronized to eliminate the effect of different levels of reproductive hormones), tissue (plasma vs. PVAT), or time of leptin injection (before vs. after endotoxin injection).

## 5. Conclusions

To conclude, acute inflammation induction modulates leptin receptor expression in thoracic PVAT in a season-dependent manner. In addition, exogenous leptin influences *IL1B* and *TNFA* gene expressions, which may moderate the inflammatory reaction progress in this examined adipose tissue, showing leptin as a significant risk factor in atherosclerosis progression. Moreover, in ewe, the sensitivity of PVAT on leptin action on proinflammatory cytokines and their receptors is dependent upon the photoperiodic condition with stronger effects stated in the SD season.

Moreover, the obtained results showing the presence of decreased sensitivity of PVAT on leptin action in PVAT of “long-day” sheep may suggest that such sheep can be an interesting model in cardiovascular system studies. This seems particularly important, knowing that rodents do not have PVAT, and pigs, which are very often used in PVAT research, do not show seasonal changes in leptin sensitivity that is specific to seasonal animals, like sheep.

## Figures and Tables

**Figure 1:**
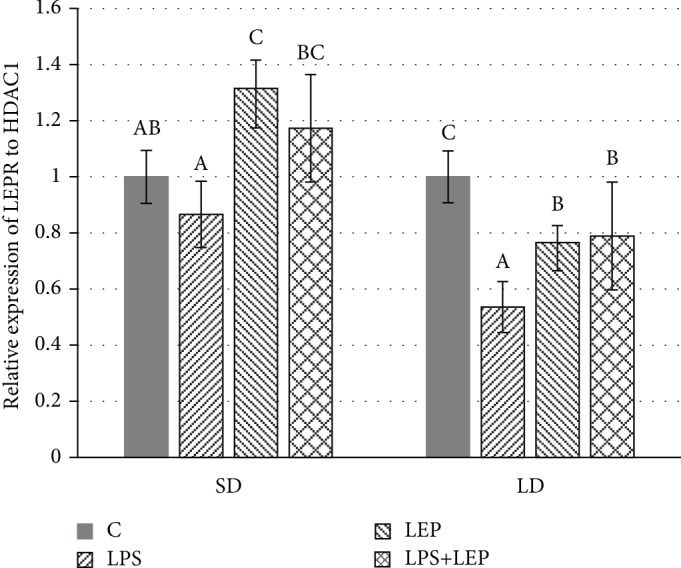
Relative gene expression of the leptin receptor (*LEPR*) in ewe's thoracic perivascular adipose tissue (PVAT) during short-day (SD) and long-day (LD) seasons. ABC: bars with different letters differ significantly according to two-way ANOVA with *post hoc* Tukey's test for each season separately, *P* ≤ 0.05, *n* = 6.

**Figure 2:**
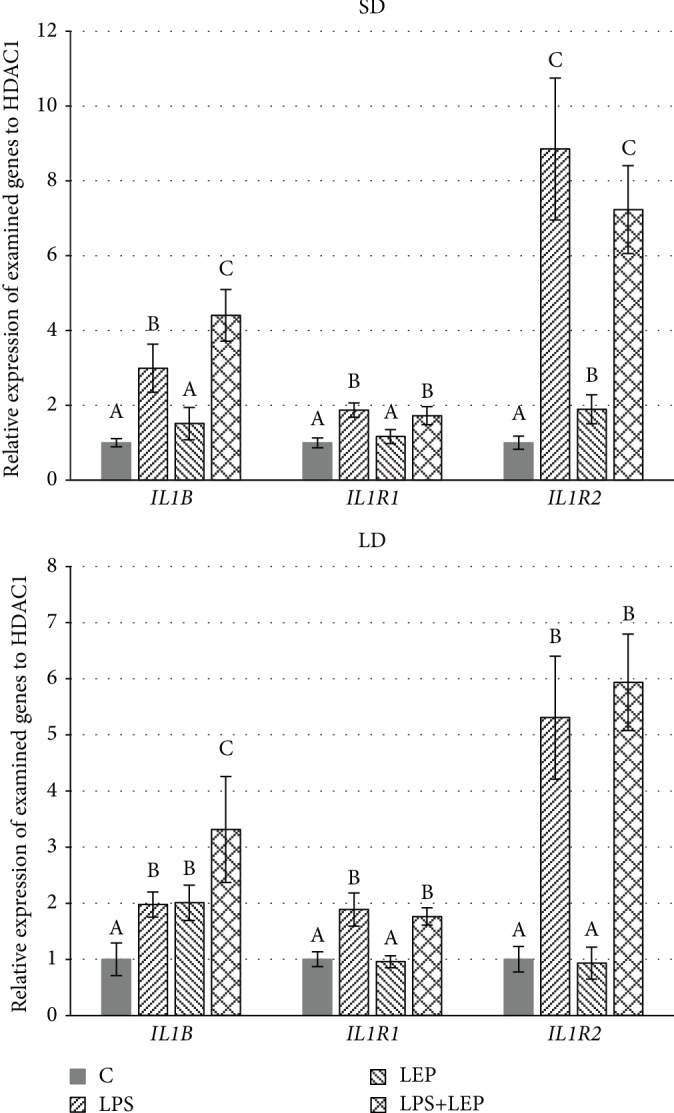
Relative gene expression of interleukin-1*β* (*IL1B*) and its receptor types 1 and 2 (*IL1R1* and *IL1R2*, respectively) in ewe's thoracic perivascular adipose tissue (PVAT) during short-day (SD) and long-day (LD) seasons. ABC: bars with different letters differ significantly according to two-way ANOVA with *post hoc* Tukey's test for each examined gene separately, *P* ≤ 0.05, *n* = 6.

**Figure 3:**
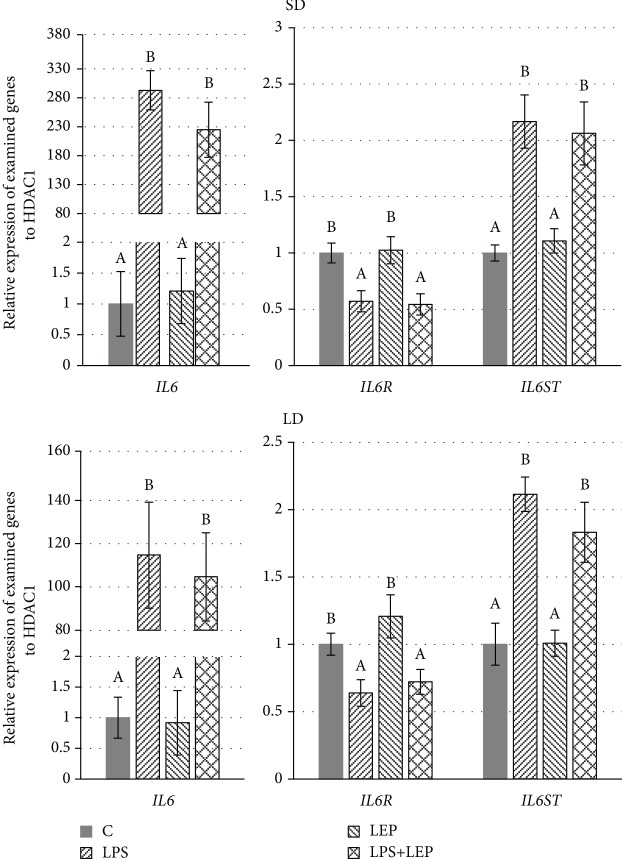
Relative gene expressions of interleukin-6 (*IL6*), its receptor (*IL6R*), and signal transducer (*IL6ST*) in ewe's thoracic perivascular adipose tissue (PVAT) during short-day (SD) and long-day (LD) seasons. ABC: bars with different letters differ significantly according to two-way ANOVA with *post hoc* Tukey's test for each gene separately, *P* ≤ 0.05, *n* = 6.

**Figure 4:**
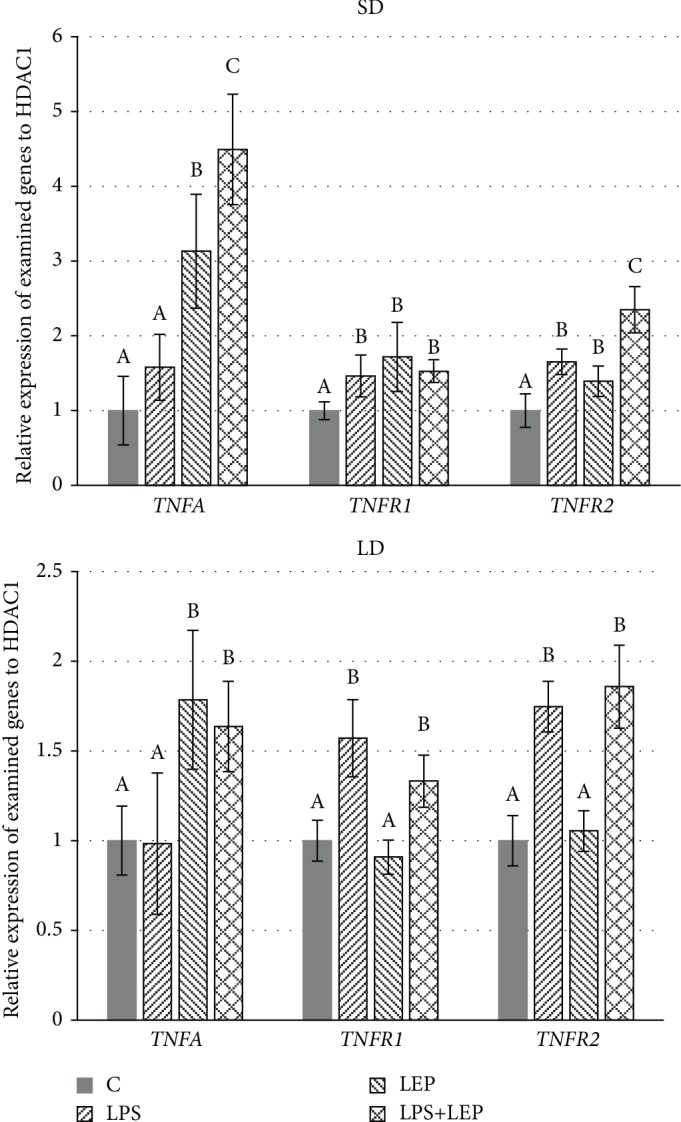
Relative gene expression of tumour necrosis factor *α* (*TNFA*) and its receptor types 1 and 2 (*TNFR1* and *TNFR2*, respectively) in ewe's thoracic perivascular adipose tissue (PVAT) during short-day (SD) and long-day (LD) seasons. ABC: bars with different letters differ significantly according to two-way ANOVA with *post hoc* Tukey's test for each gene separately, *P* ≤ 0.05, *n* = 6.

**Table 1:** Experiment scheme.

No.	Group	Number of animals	Experimental factor	LPS dose (ng/kg of body mass)	Leptin dose (*μ*g/kg of body mass)
Experiment 1: short-day period					
1	Control (C)	6	NaCl	0	0
2	LPS	6	LPS	400	0
3	LEP	6	Leptin	0	20
4	LPS+LEP	6	LPS+leptin injected 30 min after LPS	400	20
Experiment 2: long-day period					
5	Control (C)	6	NaCl	0	0
6	LPS	6	LPS	400	0
7	LEP	6	Leptin	0	20
8	LPS+LEP	6	LPS+leptin injected 30 min after LPS	400	20

**Table 2:** Genes analysed by real-time RT-PCR with Gene Bank accession numbers to gene sequence, full name, abbreviation, amplicon size and location in gene sequence, and primer characteristics.

Gene Bank acc. no.	Gene	Amplicon size (bp) (localization in the gene sequence)	Primer (forward, F; reverse, R) sequence 5′→3′	Reference
NM_001009763.1	*LEPR*: leptin receptor	123 (665 (exon 6)–787 (exon 6/7))–encoding all forms of receptor	F: CTGTGCCAACAGCCAAACT R: GTGGATCAGGCTTCACAACA	Originally designed
NM_001009465.2	*IL1B*: interleukin-1*β*	137 (373–509)	F: CAGCCGTGCAGTCAGTAAAA R: GAAGCTCATGCAGAACACCA	[24]
NM_001206735.1	*IL1R1*: IL1 receptor, type I	124 (1455–1578)	F: GGGAAGGGTCCACCTGTAAC R: ACAATGCTTTCCCCAACGTA	[25]
NM_001046210.2	*IL1R2*: IL1 receptor, type II	161 (981–1141)	F: CGCCAGGCATACTCAGAAA R: GAGAACGTGGCAGCTTCTTT	[26]
NM_001009392.1	*IL6*: interleukin-6	165 (361–525)	F: GTTCAATCAGGCGATTTGCT R: CCTGCGATCTTTTCCTTCAG	[25]
NM_001110785.3	*IL6R*: IL6 receptor	149 (288–436)	F: TCAGCGACTCCGGAAACTAT R: CCGAGGACTCCACTCACAAT	[25]
XM_012096909.2	*IL6ST*: IL6 signal transducer (glycoprotein 130)	139 (573–711)	F: GGCTTGCCTCCTGAAAAACC R: ACTTCTCTGTTGCCCACTCAG	[27]
NM_001024860.1	*TNFA*: tumour necrosis factor	153 (426–578)	F: CAAATAACAAGCCGGTAGCC R: AGATGAGGTAAAGCCCGTCA	[25]
NM_001166185.1	*TNFR1* (*TNFRSF1A*): TNF receptor, type I (TNF superfamily member 1A)	137 (310–446)	F: AGGTGCCGGGATGAAATGTT R: CAGAGGCTGCAGTTCAGACA	[25]
NM_001040490.2	*TNFR2* (*TNFRSF1B*): TNF receptor, type II (TNF superfamily member 1B)	122 (772–893)	F: ACCTTCTTCCTCCTCCCAAA R: AGAAGCAGACCCAATGCTGT	[25]
NM_001190390.1	*GAPDH*: glyceraldehyde-3-phosphate dehydrogenase	143 (135–258)	F: TGACCCCTTCATTGACCTTC R: GATCTCGCTCCTGGAAGATG	[24]
NM_001009784.2	*ACTB*: beta actin	122 (426–547)	F: GCCAACCGTGAGAAGATGAC R: TCCATCACGATGCCAGTG	[26]
XM_004005023.3	*HDAC1*: histone deacetylase 1	115 (722–836)	F: CTGGGGACCTACGGGATATT R: GACATGACCGGCTTGAAAAT	[25]

Primers designed using Primer3web version 4.0.0 (http://bioinfo.ut.ee/primer3/).

## Data Availability

The data used to support the findings of this study are available from the corresponding author upon request.
